# Frequency of Important CYP450 Enzyme Gene Polymorphisms in the Iranian Population in Comparison with Other Major Populations: A Comprehensive Review of the Human Data

**DOI:** 10.3390/jpm11080804

**Published:** 2021-08-18

**Authors:** Navid Neyshaburinezhad, Hengameh Ghasim, Mohammadreza Rouini, Youssef Daali, Yalda H. Ardakani

**Affiliations:** 1 Biopharmaceutics and Pharmacokinetic Division, Department of Pharmaceutics, Faculty of Pharmacy, Tehran University of Medical Sciences, Tehran 14176-14411, Iran; navid.pha2@gmail.com (N.N.); hengameh_ghasim@yahoo.com (H.G.); rouini@tums.ac.ir (M.R.); 2 Division of Clinical Pharmacology and Toxicology, Geneva University Hospitals, 1205 Geneva, Switzerland; 3 Faculty of Medicine, University of Geneva, 1206 Geneva, Switzerland; 4 Institute of Pharmaceutical Sciences of Western Switzerland, University of Geneva, 1211 Geneva, Switzerland

**Keywords:** CYP450 enzymes, genetic polymorphism, personalized medicine, metabolism, Iranian population

## Abstract

Genetic polymorphisms in cytochrome P450 genes can cause alteration in metabolic activity of clinically important medicines. Thus, single nucleotide variants (SNVs) and copy number variations (CNVs) in CYP genes are leading factors of drug pharmacokinetics and toxicity and form pharmacogenetics biomarkers for drug dosing, efficacy, and safety. The distribution of cytochrome P450 alleles differs significantly between populations with important implications for personalized drug therapy and healthcare programs. To provide a meta-analysis of CYP allele polymorphisms with clinical importance, we brought together whole-genome and exome sequencing data from 800 unrelated individuals of Iranian population (100 subjects from 8 major ethnics of Iran) and 63,269 unrelated individuals of five major human populations (EUR, AMR, AFR, EAS and SAS). By integrating these datasets with population-specific linkage information, we evolved the frequencies of 140 CYP haplotypes related to 9 important CYP450 isoenzymes (CYP1A2, CYP2B6, CYP2C8, CYP2C9, CYP2C19, CYP2D6, CYP2E1, CYP3A4 and CYP3A5) giving a large resource for major genetic determinants of drug metabolism. Furthermore, we evaluated the more frequent Iranian alleles and compared the dataset with the Caucasian race. Finally, the similarity of the Iranian population SNVs with other populations was investigated.

## 1. Introduction

Personalized medicine deals with the notions related to what is necessarily targeted by therapeutics and associated diagnostics, and proposes to improve the effectiveness and safety of health interventions [1]. The relationship between personalized medicine and human drug metabolizing enzymes has become a hot topic attributed to the evolution of the Human Genome Project [2]. Among all the drug metabolizing enzymes, the cytochrome P450 (CYP) superfamily plays an undeniable role and is responsible for the oxidation of almost 90% of currently used medications [3], biotransformation of other xenobiotics [4], and even endogenous compounds [5]. Modulation of CYP enzymes activity results in altered pharmacokinetics of drugs metabolized by these enzymes [6,7]. Apart from intra-individual changes in the activity of these enzymes by a number of physiological, pathological, and environmental factors [8,9,10,11,12], the expression of CYP genes can present consequential inter-individual variations in the basal expression, giving rise to diversities in the rates of drug elimination and response [13].

Pharmacogenomics investigates potential effects of inter-individual variations in the DNA sequence of distinct genes on the patients’ response to the therapeutic regimens [14,15,16]. Most genes encoding CYPs are extremely polymorphic and hold a considerable collection of single nucleotide variants (SNVs). Polymorphisms in the CYP family may have a major impact on the expected outcomes after therapeutic agent administrations [16,17]. Patients’ responses to therapeutic regimens are highly variable between individuals, resulting in inadequate efficacy of pharmacological treatments or adverse drug effects in 40–70% of patients [18]. Therefore, data of numerous studies on the intra- and inter-ethnic differences in the CYP polymorphisms suggest that the ethnicity, as a factor, can have a significant impact on the drug response [19] and the disease state of patients [20,21,22,23].

A global distribution map of the clinically important CYP alleles has been provided by several studies that demonstrates remarkable differences in the distribution of CYP alleles between populations, which could be very important in the field of personalized medicine and healthcare programs [7,17,24,25]. According to a literature review, only a few studies have evaluated the genetic polymorphisms of some major cytochrome P450 enzymes in the Iranian population [26,27,28,29]. However, the significance of these studies is limited due to the relatively small sample sizes and the focus on a few, non-standardized panels of specific variants of interest.

The human genome project achievement in 2003 was a crucial landmark in the field of genetics. After that, several human genome variation databases, such as International HapMap, NHLBI Exome, 1000 Genomes, and finally Genome Aggregation databases, were made available worldwide by introducing new technologies such as next generation sequencing (NGS) [17]. Such databases provide a completed analysis of the genetic variation and interethnic diversity of CYP alleles across a specific population, enabling the comparison of results with other ethnics of the world.

Although available databases have been playing a crucial role in interpreting genetic variations, many ethnic groups are not represented in these records. As a lot of genome variations in human are ethnicity-specific, having representatives from all different ethnic groups can greatly help to build a complete picture of genetic variations in the databases. On the other hand, the lack of ethnic-specific data may put some members of those populations in danger of discrimination by depriving them of the benefits of some new medical advances.

Keeping this point in mind, a whole exome sequencing on 800 individuals from eight major ethnic groups in Iran was established in the Iranome database. Available online: http://www.iranome.ir (accessed on 14 August 2021) in collaboration with Iranian and Canadian researchers. These individuals represent more than 80 million Iranians and nearly half a billion people living in the Middle East, a region with a rapid population growth expectation and to date, rare information about these ethnic groups has been provided in human genomic diversity databases.

In this study, therefore, the genetic polymorphisms of the major cytochrome P450 enzymes in Iranian population, based on a literature review and Iranome database, were compared with other related data in this field, based on other available human genome variation databases. The resulting data of this meta-analysis, to our knowledge, are the most extensive overview of CYP allele distributions in Iranian population published to date and provide substantial information for the guidance of population-specific genotyping strategies in pharmacokinetic studies and eventually in personalized medicine.

## 2. Methods

### 2.1. Allele Frequency Data

In the present study, the prevalence of 140 variants related to the genes of nine Cytochrome P450 enzymes were evaluated accounting for more than 75% of phase I drug metabolism in humans. The Iranian population (IRN) (a total number of 800 people—100 individuals from each of the eight main ethnic groups of the country) we studied using the Iranome database. Available online: http://www.iranome.ir (accessed on 14 August 2021) The obtained results were compared with the five major populations of the world (European (EUR) Genomes, African (AFR) Genomes, Latino/Admixed American (AMR) Genomes, East Asian (EAS) Genomes, and South Asian (SAS) Genomes) using the website (https://gnomad.broadinstitute.org (accessed on 14 August 2021)). Moreover, a comparison was made with the Caucasian race through the related papers (Table 1).

### 2.2. Allele Nomenclature and Definitions

CYP star (*) alleles were defined according to the Human CYP Allele Nomenclature Database. Available online: http://www.pharmvar.org (accessed on 14 August 2021) and SNP database. Available online: http://www.SNPedia.com (accessed on 14 August 2021), and citations were presented for the sources of references describing the functional characterization of the evaluated alleles. Similarity of high-frequency alleles (SNPs) in Iranian population with other populations has been demonstrated in Table 2. All the sub-alleles and the variants affecting the functionality of the enzyme were evaluated as indicated in Table 3 for Caucasians versus Iranian population and in Tables S1–S9 (Supplemental Material) for the five major populations in the world in comparison with Iranian population.

In this regard, common alleles were defined as having minor allele frequency (MAF) >1%, whereas variants or alleles with an allelic frequency from 0.1% to 1% were defined as rare, and those with a frequency less than 0.1% were considered zero.

## 3. Results and Discussion

To date, several researchers have reported the frequency of different CYP alleles in selected populations around the world. However, most of the studies have focused only on a specified groups of single nucleotide variants in each gene and/or studied the prevalence of the alleles in a limited sample size of those populations [30,31,32,33]. Recently, an extensive meta-analysis of 173 reports has revealed the spectrum of allele frequencies and predicted functional consequences across major populations for 12 CYP450 enzymes [17].

There are also several studies on evaluating the polymorphisms of most important CYP450 enzymes in IRNs with similar mentioned limitations (investigating some specific CYP450 enzymes, examining only limited number of alleles, and/or recruiting a limited number of participants). Moreover, relying on the results of these studies can be problematic due to the different underlined genotyping strategies and designed assay panels [28,34,35,36].

Given all the points mentioned above, the Iranome Browser consisting of a whole exome sequencing of 800 individuals from eight major ethnic groups of Iran was considered in this study as the main source of data to allow an accurate analysis of the genetic polymorphisms of the major cytochrome P450 enzymes across Iranian population. In the next step, extracted data were compared to other ethnics of the world based on a literature review and other available databases.

In this regard, 140 variants related to nine important CYP450 isoenzymes (CYP1A2, CYP2B6, CYP2C8, CYP2C9, CYP2C19, CYP2D6, CYP2E1, CYP3A4, and CYP3A5) were analyzed and will be discussed in more detail, respectively.

### 3.1. Cytochrome P450 1A2 (CYP1A2)

This enzyme (also called phenacetin O-deethylase) metabolizes a wide range of endogenous (e.g., steroids and arachidonic acid) and exogenous (clozapine, duloxetine, tizanidine, melatonin, etc.) compounds. A substantial inter-subject variation (10–200 times) has been reported in the protein expression and the activity of this enzyme, approximately 35–75% of which have been attributed to genetic factors. In this regard, more than 15 different variants and a series of subvariants (*1B to *16) have been identified for the CYP1A2 gene [37].

**Figure 1:**
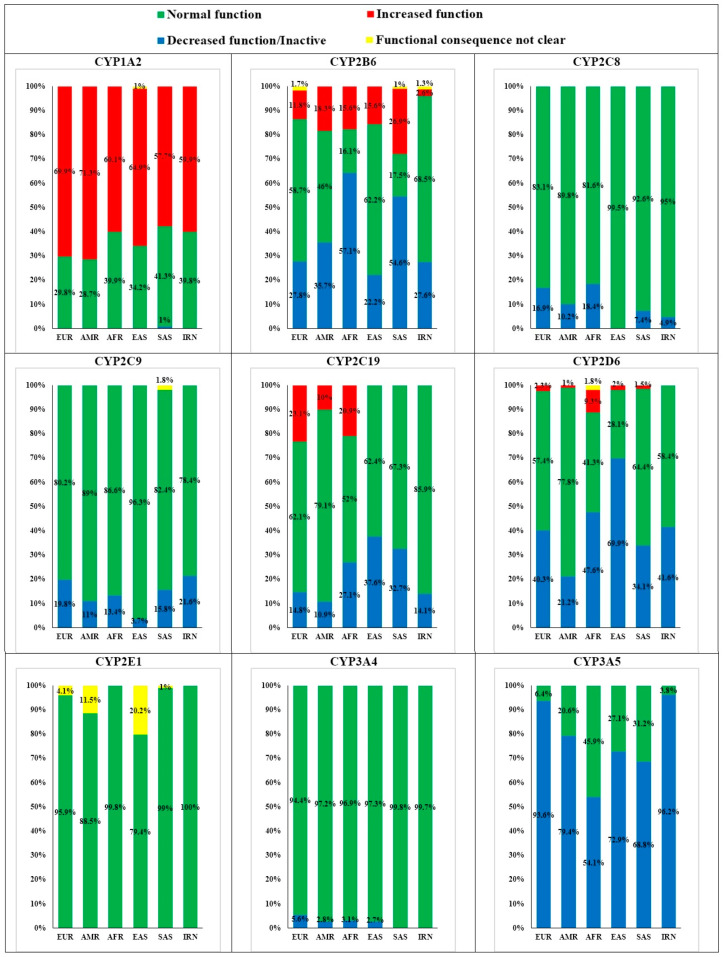
CYP1A2, CYP2B6, CYP2C8, CYP2C9, CYP2C19, CYP2D6, CYP2E1, CYP3A4, and CYP3A5 alleles frequencies in five major populations compared to the Iranian population.

Thirteen clinically important variants of CYP1A2 were assessed in the present study. Analysis of the acquired data in IRNs showed that about 39.8% of the population had *1 allele with normal activity, which was very similar to the AFRs in terms of prevalence (Figure 1).

**Figure 2:**
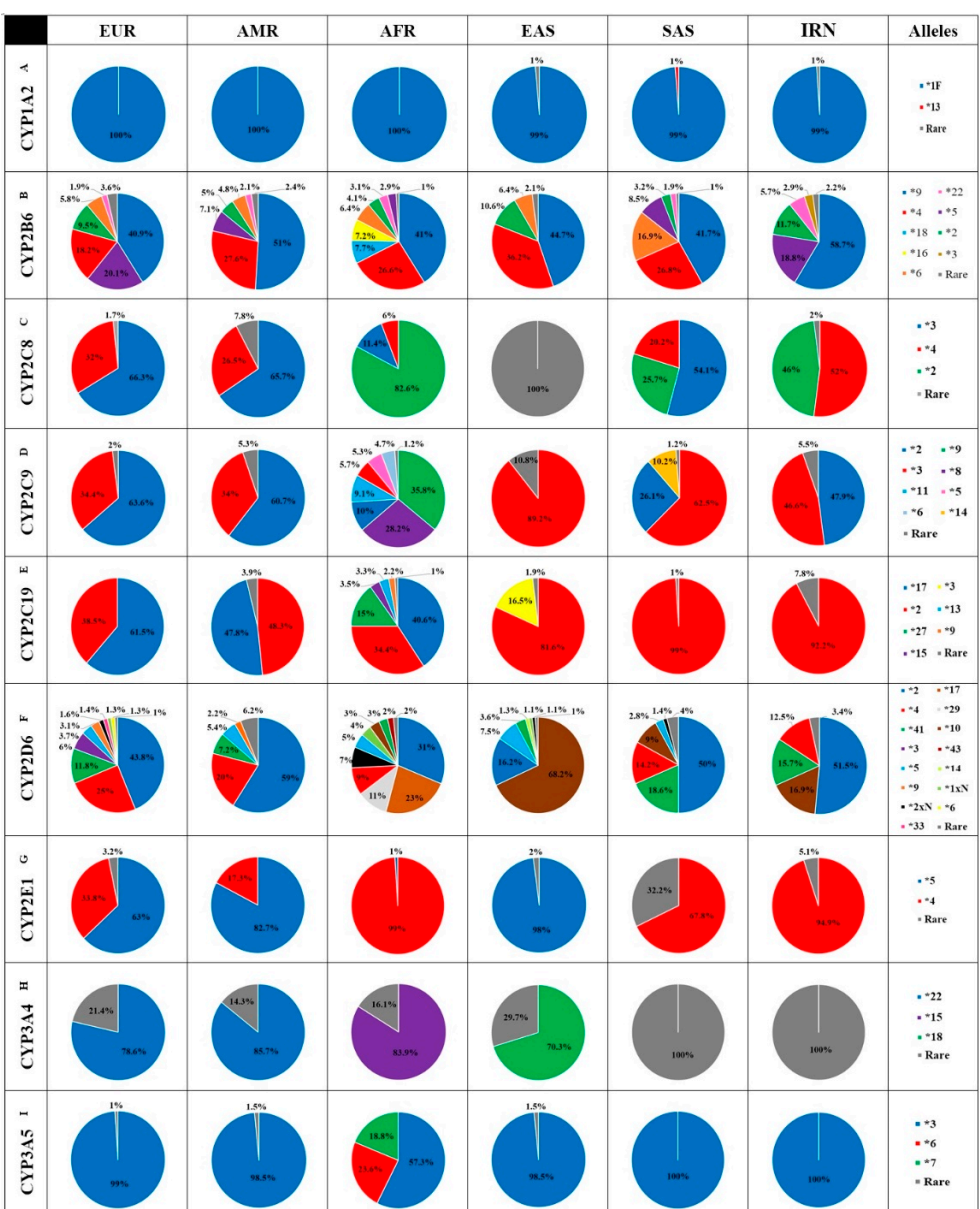
Distribution of the relative contributions of 9 major cytochrome P450 (CYP) alleles in Iranian, European, Latino/Admixed American, African, East Asian and South Asian populations. Pie charts showing the relative contribution of common variants in CYP1A2 (**A**), CYP2B6 (**B**), CYP2C8 (**C**), CYP2C9 (**D**), CYP2C19 (**E**), CYP2D6 (**F**), CYP2E1 (**G**) CYP3A4 (**H**), and CYP3A5 (**I**) in five major populations compared to Iranian population. Only variants with a minor allele frequency in the respective population above 1% are shown, whereas all other variants analyzed in this study are summarized as “rare” (grey).

Examining the important mutant variants indicates that approximately about 60% of IRNs carry *1F (rs762551) variant of CYP1A2. Homozygous carriers of *1F alleles (*1F/*1F genotype) are fast metabolizers. Interestingly, the inducibility characteristics of CYP1A2, especially the CYP1A2*1F allele, are higher than that of other CYPs [38]. Smoking is known as the most important factor among CYP1A2 inducers [39]. Compared to other populations, the prevalence observed for the *1F allele was similar to those reported for the AFRs (60.1%) and SASs (57.7%) (Table S1 and Figure 2A).

The prevalence of this mutant was reported to be about 73.3% by Myrand SP et al. (Table 3) [40]. Surprisingly, no similarity was found between IRNs and CAUCs in terms of the frequency of this variant.

The other alleles, with decreased activity or inactivity, showed a prevalence of less than 1% (MAF).

### 3.2. Cytochrome P450 2B6 (CYP2B6)

Several medications (e.g., bupropion, cyclophosphamide, efavirenz, nevirapine, and methadone) are primarily metabolized by CYP2B6.

The expression of this enzyme is highly variable (intra- and inter-individual variations) due to genetic polymorphisms and nongenetic factors (e.g., inducibility and irreversible inhibition) in human liver.

The CYP2B6 gene is one of the most polymorphic CYP genes in humans, with many of the identified variants affecting the catalytic activity of the enzyme. In this regard, 25 different variants of CYP2B6 with clinical importance were reported in several studied populations (Table S2). Among these, nine mutants (*2, *3, *4, *5, *6, *9, *16, *18, and *22) showed the highest frequencies with considerable differences between populations.

Considering the major variants, about 68.5%, 27.6%, and 2.6% of IRNs had alleles with normal activity, low activity or inactivity, and high activity, respectively, and 1.3% showed alleles with uncertain functions. Therefore, no similarities were found between the IRNs and the other studied populations in terms of the frequency of normal and mutant variants (Figure 1).

Considering the mutant alleles, CYP2B 6*9/*5/*2/*22, and *3 alleles had the highest values of prevalence (about 26.6%, 8.5%, 5.3%, 2.6% and 1.3%, respectively) in IRNs. Other variants of this cytochrome were considered as MAF. Compared to other studied populations, the highest similarities in terms of prevalence were observed in alleles *9 and *2 with EURs and Caucasians (26.6% vs. 24% and 28.6% for *9, and 5.3% VS 6.6% and 5.3% for *2 in EURs and Caucasians, respectively) [41]. The prevalence of the *5 allele in IRNs was remarkably the same as SASs and Caucasians (8.5% vs. 8% and 10.9% in SASs and Caucasians, respectively), while allele *22 was most similar to AFRs and Caucasians (2.6% vs. 2.8% and 2.4% in AFRs and Caucasians, respectively). Surprisingly, the prevalence of *3 allele showed no resemblance to the other studied populations (Table S2 and Figure 2B).

The CYP2B6*9, the most abundant CYP2B6 mutant alleles in IRNs, decreases the activity of the enzyme. Interestingly, although CYP2B6*5 apparently results in significantly reduced expression levels of CYP2B6 in vitro, no changes in the metabolism of efavirenz may suggest increased specific activity of the gene product, which compensates for the reduced expression levels [42]. In agreement with these in vitro findings, no effect of CYP2B6*5 was observed on efavirenz pharmacokinetics in vivo [46].

Among the most frequent mutant alleles in IRNs, the CYP2B6*22 results in increased functionality of the enzyme, which shows a prevalence of about 2.6% in IRNs.

### 3.3. Cytochrome P450 2C8 (CYP2C8)

High levels of CYP2C8 are found in human liver [47] and contribute to the metabolism of several important drugs, including paclitaxel, all-trans retinoic acid, verapamil, rosiglitazone, montelukast, pioglitazone, amiodarone, and repaglinide [48]. Some evidence indicates that CYP2C8 plays a physiological role in the metabolism of arachidonic acid. However, recent reports show that CYP2C9 may be the main isoform taking part in this process [49]. This enzyme may also play a role in the bioactivation of important toxicological compounds such as benzo[a]pyrene and parathion [50]. CYP2C8 shares significant sequence homology with other members of the CYP2C family, especially CYP2C9, but exhibits relatively little overlap of substrate specificity. For instance, CYP2C8 and CYP2C9 are the most relevant enzymes involved in the metabolism of non-steroidal anti-inflammatory drugs (e.g., ibuprofen), and the potential role of the polymorphism of these two enzymes in the pharmacokinetics and as a result of that in the gastrointestinal bleeding risk during the use of such drugs is undeniable [51].

Comparing the activity of the most frequent variants, it was specified that the IRNs had 95% of *1 allele with normal activity and 5% of low-active or inactive alleles. The results were similar to the SASs (Figure 1).

Based on investigating the prevalence of mutant variants of this enzyme, *4 and *2 alleles were observed to have the highest values (2.6% and 2.3%, respectively) within the IRNs. Thus, totally about 5% of IRNs are carrying these two alleles, which results in decreased activity of this enzyme. All the other alleles of the cytochrome were considered as MAF. The highest similarity was observed in the prevalence of alleles with AMRs for *4 allele (2.6% vs. 2.7%), while *2 allele showed no similarity with the other studied populations (Table S3 and Figure 2C) [44].

### 3.4. Cytochrome P450 2C9 (CYP2C9)

CYP2C9 is the most abundant isoform of the CYP2C subfamily that is involved in about 20% of total hepatic CYPs [52]. This enzyme contributes to the metabolism of various drugs such as nonsteroidal anti-inflammatory drugs, s-warfarin, anti-diabetic drugs, phenytoin, losartan, and fluoxetine. CYP2C9*2 and CYP2C9*3 polymorphisms are found in approximately 85% of poor metabolizers (PMs), and the allele frequencies vary from 5 to 13% in CAUCs. It seems that the effect of CYP2C9 *2 on the activity of enzymes is less than that of CYP2C9*3 [53,54]. Nevertheless, both alleles reduce the metabolic activity of about 10 times as different as the wild type (CYP2C9*1/*1). The (CYP2C9*3/*3) is considered as the mutant allele with minimum activity.

Based on comparing the prevalence of 22 clinically important variants, it was found that about 78.4% of the subjects showed *1 allele with normal activity and of 21.6% had alleles with reduced or no activity, which was similar to SASs in terms of prevalence of the active form. Among the individuals representing mutant alleles, the highest values of prevalence were found for *2 (10.5%) and *3 (10.2%) alleles. All the other alleles of this cytochrome showed a prevalence of less than 1% (Table S4, Figure 2D).

Although the IRNs had no similarities with the other studied populations in terms of *2 allele prevalence, the prevalence of *3 allele was similar to SASs (10.2% vs. 11%).

According to data provided by Myrand SP et al., the prevalence levels of *2 and *3 alleles in the CAUCs were 13.3% and 5.3%, respectively [40], which shows no similarity to the IRNs (Table 3).

### 3.5. Cytochrome P450 2C19 (CYP2C19)

CYP2C19 is an important member of the CYP450 superfamily, playing a basic role in the metabolism of approximately 10% of commonly prescribed drugs such as proton pump inhibitors, antipsychotics, antidepressants, and clopidogrel [55].

Similar to many other CYP450 superfamily members, inter-individual differences in the CYP2C19 activity can be explained reasonably by genetic polymorphisms [56,57]. To date, at least 34 variants and several subvariants have been identified within the CYP2C19 gene. Due to the importance of clopidogrel bioactivation, especially through this enzyme, many studies have evaluated the correlation between genetic polymorphisms and drug resistance. Until 2006, studies carried out on CYP2C19 genetic polymorphisms (involving CYP2C19*2 and CYP2C19*3 alleles) predicted the PM and IM phenotypes. The introduction of the ultra-rapid metabolizer (UM) phenotype for the CYP2C19 enzyme was originated from the discovery of a new allele called CYP2C19*17.

By investigating 19 important variants of this enzyme in IRNs, about 85.9% of subjects had the wild type allele with normal activity and 14.1% had mutant alleles with reduced or no activity. The results were not similar to the other populations studied in terms of the prevalence of the wild type (Figure 1).

Among the individuals representing mutant alleles, only CYP2C19*2 allele was found to cause a reduction in the activity of enzymes in IRNs (13.1%). All the other variants of this cytochrome were considered as MAF. A comparison of the prevalence of mutant alleles with the other major studied populations showed the similarity of the IRNs to the EURs and CUACs for *2 allele (14.1 vs. 14.6% and 13.6%) (Table S5 and Figure 2E) [40].

Surprisingly, although some studies reported the presence of *17 allele in IRNs, no subject with *17 allele was found among the 800 subjects in the Iranom project (Iranome database. Available online: http://www.iranome.ir (accessed on 14 August 2021)).

### 3.6. Cytochrome P450 2D6 (CYP2D6)

Although CYP2D6 involves only about 1.5% of total hepatic CYPs, it contributes to the metabolism of approximately 25% of all medications including tricyclic antidepressants, selective serotonin reuptake inhibitors, neuroleptic agents (antipsychotics), β-blockers, antiarrhythmic drugs, tamoxifen, and opioids [15,16].

The CYP2D6 gene is highly polymorphic with more than 80 allelic variants, based on the reports on the webpage of the CYP450 Allele Nomenclature Committee. Available online: http://www.pharmvar.org (accessed on 14 August 2021). Moreover, it is necessary to determine the clinical effects of most of these genetic variants. While CYP2D6*3/*4/*5/*6 variants often predict poor metabolizers (PMs) and intermediate metabolizers (IMs), *2 allele is mostly found in multiple CYP2D6 genes and predicts the extensive metabolizers (EMs). While PMs have less potential to bio-transform active metabolites after prescribing prodrugs, the UMs may show harmful side effects [58].

Similar to CYP2B6, variants of CYP2D6 had the highest variety with a prevalence of more than 1% within the IRNs. Twenty-two variants of CYP2D6 with clinical importance were assessed in the present study. Examining the important variants of CYP2D6 within the IRNs, prevalence values of 58.4% and 41.6% were found, respectively, for the wild type and for the mutant alleles with low or zero activity, which was similar to that of EURs (Figure 1). Among the individuals with low or inactive alleles of this cytochrome, the highest values of prevalence were found for CYP2D6*2 (47%), *10 (15.1%), *41 (14%), and *4 (11.2%) alleles, respectively. The values associated with the other alleles were less than 1%.

According to the collected data, (Table S6 and Figure 2F), the prevalence of *41 allele in IRNs was similar to that of SASs (13.5% vs. 14.0%) and that of *4 allele resembled SASs and AMRs (11.2% vs. 11.9% and 10.3%, respectively). No similarity was observed between the prevalence of * 2 and * 10 alleles in IRNs and the other populations (Table S6 and Figure 2F). Surprisingly, the IRNs had no similarity to CAUCs in terms of the prevalence of CYP2D6 mutant variants (Table 3) [40].

### 3.7. Cytochrome P450 2E1 (CYP2E1)

CYP2E1 enzyme has been of interest due to its role in the metabolism and bioactivation of many low molecular weight compounds including ethanol and acetone, acetaminophen, isoniazid, tobacco, fluorinated anesthetics, and many procarcinogens (e.g., benzene, N-Nitrosodimethylamine, and styrene) [59,60]. The CYP2E1 gene shows several genetic polymorphisms, possibly leading to interindividual variability in drug response, drug–drug, drug–xenobiotic interactions, and susceptibility to chemical-induced diseases [61].

Based on assessing four clinically important alleles of CYP2E1 in IRNs, *4 allele had the highest prevalence (5.6%) among the alleles of CYP2E1. The other mutant alleles were considered as MAF. In terms of *4 prevalence, no similarity was found to the other populations studied (Table S7 and Figure 2G). It should be noted that the activity of the above-mentioned allele is reported to be normal. Thus, almost 100% of the observed alleles in IRNs had normal activity, similar to those of SASs and AFRs (Figure 1). According to a literature review, no report was found on *4 allele frequency in the CAUCs (Table 3).

### 3.8. Cytochrome P450 3A4 (CYP3A4)

CYP3A4 is the most abundant hepatic and intestinal phase I enzyme that metabolizes approximately 50% of marketed drugs [62].

To date, more than 40 single nucleotide polymorphisms (SNPs) of CYP3A4/5 have been reported to the Human P450 Allele Nomenclature Committee. However, most functional SNPs in CYP3A4 are rare and involve a small fraction of interindividual variations.

Recently, a new functional SNP located in CYP3A4 intron 6 (CYP3A4*22) has been found to be correlated with the enzyme activity in the human liver [63,64]. CYP3A4*22 allele has been used as an important biomarker to recognize a decrease in CYP3A4 drug metabolism and is reportedly correlated with the decreased dose needed for optimal drug therapy associated with simvastatin, tacrolimus, and cyclosporine [62].

In this study, 19 clinically important variants of CYP3A4 were assessed in IRNs, where a prevalence of almost 99.7% was indicated for *1 allele with normal activity, similar to those of all the other studied populations (Figure 1). None of the CYP3A4 alleles had a prevalence of more than 1% in the IRNs, remarkably the same as SASs (Table S8 and Figure 2H).

### 3.9. Cytochrome P450 3A5 (CYP3A5)

CYP3A5 enzyme is the main extra-hepatic isoform in the CYP3A gene family and, along with CYP3A4, contributes to the metabolism of more than 50% of clinically used drugs. These two isoenzymes exhibit a significant overlap in substrate specificity [7]. In many cases, CYP3A5 is more susceptible to CYP3A-metabolized drugs such as midazolam and tacrolimus than CYP3A4.

The CYP3A5*3 polymorphism is widely recognized in Europeans, and the homozygous variant is strongly correlated with decreased enzymatic activity. Conversely, the CYP3A5*1 allele is correlated with high CYP3A5 expression. The carriers of the CYP3A5*1 allele were determined as CYP3A5 receptors and showed an increase in the metabolism of CYP3A4/5 substrates [65,66].

Examination of clinically important variants of CYP3A5 (seven variants) within the IRNs indicated a prevalence of 3.8% for *1 allele with normal activity, while about 96.2% of the population had inactive alleles or with decreased activity similar to EURs (Figure 1).

Among individuals with mutant alleles, *3 allele had the highest prevalence (96.1%), similar to that of EURs (93%). All the other examined alleles were MAF (Table S9 and Figure 2I). According to the report of Myrand SP et al., the prevalence of *3 allele was 95.9% in CAUCs, showing similarity to the IRNs (Table 3) [40].

## 4. Conclusions

Although many previous studies on the polymorphisms of major CYP isoforms in the Iranian population were based on the proximity of this population to the Caucasians, the results of this study indicate drastic genetic differences between IRNs and Caucasians, and even a major significant difference between the Iranian population and other ethnic groups (European, Latino/Admixed American, African, East Asian and South Asian populations) in terms of CYP allele frequencies. Therefore, it can be concluded that the Iranian population should be considered as a specific ethnic in future genotypic and phenotypic studies related to the activity of CYP enzymes. Availability of more ethnicity-specific data can be very helpful to build a complete picture of genetic variations, leading to more accurate dose adjustments in patients and thus faster progress in the field of personalized medicine. Moreover, in connection with the study of the activity of CYP450 enzymes, it is important to study the phenotype of these enzymes along with their genotype, because numerous studies have shown that, despite the normal genetic status of these enzymes in individuals, due to some environmental factors, including disease, diets, behavioral habits and specific lifestyles, phenotypes and activities of these enzymes change. Therefore, it seems inevitable to study the correlation of genotype and phenotype (e.g., phenotyping using cocktail approach) of these enzymes simultaneously for more confidence while performing analyzing of CYP450 enzymes and higher accuracy in personalized medicine studies.

## Figures and Tables

**Table 1:** Number of Genomes sequenced in Iranian population compared to 5 major populations and Caucasians.

Population	Description	Genomes
**IRN**	Iranian Based on Iranome database. Available online: http://www.iranome.ir (accessed on 14 August 2021)	800
**AMR**	Latino/Admixed American Based on Genome database. Available online: https://gnomad.broadinstitute.org (accessed on 14 August 2021)	6835
**EUR**	Non-Finnish European Based on Genome database. Available online: https://gnomad.broadinstitute.org (accessed on 14 August 2021)	32,299
**AFR**	Based on Genome database. Available online: https://gnomad.broadinstitute.org (accessed on 14 August 2021)	21,042
**EAS**	East Asian Based on Genome database. Available online: https://gnomad.broadinstitute.org (accessed on 14 August 2021)	1567
**SAS**	South Asian Based on Genome database. Available online: https://gnomad.broadinstitute.org (accessed on 14 August 2021)	1526
**CAUC**	Caucasian (based on literature review)	~100–250

**Table 2:** Similarity of high-frequency CYP alleles in the Iranian population with other populations.

CYP450 Enzyme	Allele	SNP ID	Frequency in IRNs	Similarity with Five Major Populations (Freq.)
**CYP1A2**	*1F	(rs762551)	59.9%	AFR (60.1%), SAS (57.7%)
**CYP2B6**	*9	(rs3745274)	26.6%	EUR (24%), Cauc. (28.6%)
	*5	(rs3211371)	8.5%	SAS (8%)
	*2	(rs8192709)	5.3%	EUR (5.6%), Cauc. (5.3%)
	*22	(rs34223104)	2.6%	AFR (2.8%), Cauc. (2.4%)
	*3	(rs45482602)	1.3%	No similarity
**CYP2C8**	*4	(rs1058930)	2.6%	AMR (2.7%)
	*2	(rs11572103)	2.3%	No similarity
**CYP2C9**	*2	(rs1799853)	10.5%	No similarity
	*3	(rs1057910)	10.2%	SAS (11%)
**CYP2C19**	*2	(rs4244285)	13.1%	EUR (14.6%), Cauc. (13.6%)
**CYP2D6**	*2	(rs16947, rs1135840)	47%	No similarity
	*10	(rs1065852, rs1135840)	15.1%	No similarity
	*41	(rs28371725)	14%	SAS (13.5%)
	*4	(rs3892097)	11.2%	AMR (11.9%)
**CYP2E1**	*4	(rs6413419)	5.6%	No similarity
**CYP3A4**	-	-	-	-
**CYP3A5**	*3	(rs776746)	96.1%	EUR (93%), Cauc. (95.5%)

**Table 3:** Prevalence of frequent alleles related to 9 CYP450 genes in Iranian population compared to Caucasian race.

CYP450 Enzyme	Allele	SNP ID	Frequency in IRNs	Frequency in CAUC
**CYP1A2**	*1F	(rs762551)	59.9%	73.7% [40]
**CYP2B6**	*9	(rs3745274)	26.6%	28.6% [41]
	*5	(rs3211371)	8.5%	10.9% [41]
	*2	(rs8192709)	5.3%	5.3% [41]
	*22	(rs34223104)	2.6%	2.4% [42]
	*3	(rs45482602)	1.3%	<1% [41]
**CYP2C8**	*4	(rs1058930)	2.6%	7.5% [43]
	*2	(rs11572103)	2.3%	<1% [44]
**CYP2C9**	*2	(rs1799853)	10.5%	13.3% [40]
	*3	(rs1057910)	10.2%	5.6% [40]
**CYP2C19**	*2	(rs4244285)	13.1%	13.6% [40]
**CYP2D6**	*2	(rs16947, rs1135840)	47%	32.8–52.5% [40]
	*10	(rs1065852, rs1135840)	15.1%	19.6% [40]
	*41	(rs28371725)	14%	9.6% [45]
	*4	(rs3892097)	11.2%	18.2% [40]
**CYP2E1**	*4	(rs6413419)	5.6%	NA
**CYP3A4**	-	-	-	-
**CYP3A5**	*3	(rs776746)	96.1%	95.5% [40]

## Data Availability

Not applicable.

## Supplementary Materials

The following are available online at https://www.mdpi.com/article/10.3390/jpm11080804/s1, Table S1: Important variant and allele frequencies of the human CYP1A2 gene, Table S2: Important variant and allele frequencies of the human CYP2B6 gene, Table S3: Important variant and allele frequencies of the human CYP2C8 gene, Table S4: Important variant and allele frequencies of the human CYP2C9 gene, Table S5: Important variant and allele frequencies of the human CYP2C19 gene, Table S6: Important variant and allele frequencies of the human CYP2D6 gene, Table S7: Important variant and allele frequencies of the human CYP2E1 gene, Table S8: Important variant and allele frequencies of the human CYP3A4 gene, Table S9: Important variant and allele frequencies of the human CYP3A5 gene.
