# Supplementary material for: Frequency of Important CYP450 Enzyme Gene Polymorphisms in the Iranian Population in Comparison with Other Major Populations: A Comprehensive Review of the Human Data
Source: J Pers Med. 2021 Aug 18;11(8):804. doi: 10.3390/jpm11080804 (PMC8401584; doi:10.3390/jpm11080804)
Supplement: Supplementary file 1 [file jpm-11-00804-s001.zip › jpm-1270394-supplementary.pdf]

Table S1. Important variant and allele frequencies of the human CYP1A2 gene

Important variant and allele frequencies of the human *CYP1A2* gene									
			Allele frequencies in indicated populations, %						
**Allele**	**Defining variants**	**Variant type**	**EUR**	**AMR**	**AFR**	**EAS**	**SAS**	**IRN**	**Functional consequence**
***1**			29.8	28.7	39.9	34.2	41.3	39.8	Normal
***1F**	rs762551	5' UTR	69.9	71.3	60.1	64.9	57.7	59.9	Increased
***1K**	rs12720461	5' UTR	0.2	0	0	0	0.3	0	Decreased
***2**	rs56160784	(F21L)*	0	0	0	0.2	0	0	Not clear
***3**	rs56276455	(D348N)*	0	0	0	0	0	0	Decreased
***6**	rs28399424	(R431W)*	0.1	0	0	0	0	0	Inactive
***7**	rs56107638	**	0	0	0	0	0	0	Decreased
***8**	rs72547517	(R456H)*	0	0	0	0	0	0	Decreased
***9**	rs138652540	(T83M)*	0	0	0	0	0	0	Not clear
***10**	rs72547512	(E168Q)*	0	0	0	0	0	0	Not clear
***11**	rs72547513	(F186L)*	0	0	0	0	0	0	Decreased
***13**	rs35796837	(G299S)*	0	0	0	0.4	0.7	0.3	Not clear
***14**	rs45486893	(T438I)*	0	0	0	0.3	0	0	Not clear
***16**	rs72547515	(R377Q)*	0	0	0	0	0	0	Decreased

*: Missense
**: Splicing defect

Table S2. Important variant and allele frequencies of the human CYP2B6 gene

Important variant and allele frequencies of the human *CYP2B6* gene									
			Allele frequencies in indicated populations, %						
**Allele**	**Defining variants**	**Variant type**	**EUR**	**AMR**	**AFR**	**EAS**	**SAS**	**IRN**	**Functional consequence**
***1**	None		41.3	38.5	9.8	57.6	6.5	54.7	Normal
***2**	rs8192709	(R22C)*	5.6	3.1	3.7	4.5	3.0	5.3	Normal
***3**	rs45482602	(S259R)*	0.2	0.1	0	0	0.2	1.3	Not clear
***4**	rs2279343	(K262R)*	10.7	17	24	15.4	25.1	0	Increased
***5**	rs3211371	(R487C)*	11.8	4.4	2.6	0.1	8	8.5	Normal
***6**	rs2279343<br>rs3745274	(K262R)*<br>(Q172H)*	3.4	3	5.8	2.7	15.8	0	Decreased
***7**	rs2279343<br>rs3745274<br>rs3211371	(K262R)*<br>(Q172H)*<br>(R487C)*	0	0	0	0	0	0	Decreased
***8**	rs12721655	(K139E)*	0.4	0.1	0	0	0	0	Inactive
***9**	rs3745274	(Q172H)*	24	31.4	37	19	38.9	26.6	Decreased
***10**	rs8192709<br>rs34883432	(R22C)*<br>(Q21L)*	0	0.3	0	0	0.1	0	Not clear
***11**	rs35303484	(M46V)*	0.3	0.1	0	0	0.2	0.6	Not clear
***12**	rs36060847	(G99E)*	0	0	0	0	0	0	Inactive
***13**	rs2279343<br>rs3745274<br>rs12721655	(K262R)*<br>(Q172H)*<br>(K139E)*	0	0	0	0	0	0	Inactive
***14**	rs35773040	(R140Q)*	0.5	0	0	0	0.3	0.3	Not clear
***15**	rs35979566	(I391N)*	0.7	0.1	0.2	0	0	0.1	Not clear
***16**	rs2279343<br>rs28399499	(I328T)*	0	0.3	6.5	0	0	0	Decreased
***17**	rs33973337<br>rs33980385<br>rs33926104	(T26S)*<br>(D28G)*<br>(R29T)*	0	0	0	0	0	0	Normal
***18**	rs28399499	(I328T)*	0	0.3	7	0	0	0	Decreased
***19**	rs34826503	(R336C)*	0	0	0.2	0	0	0	Inactive
***20**	rs36056539	(T168I)*	0	0	0.2	0	0	0	Decreased
***21**	rs35010098	(P428T)*	0	0	0	0	0	0	Not clear
***22**	rs34223104	Regulatory	1.1	1.3	2.8	0.2	1.8	2.6	Increased
***23**	rs3211369	(M459V)*	0	0	0	0	0	0	Not clear
***26**	rs2279343<br>rs3745274<br>rs3826711	(K262R)*<br>(Q172H)*<br>(P167A)*	0	0	0	0.5	0	0	Decreased
***27**	rs36079186	(M198T)**	0	0	0.2	0	0.1	0	Not clear
***28**	rs34097093	(R378X)	0	0	0	0	0	0	Inactive

*: Missense
**: Stop-gain

Table S3. Important variant and allele frequencies of the human CYP2C8 gene

Important variant and allele frequencies of the human *CYP2C8* gene									
			Allele frequencies in indicated populations, %						
**Allele**	**Defining variants**	**Variant type**	**EUR**	**AMR**	**AFR**	**EAS**	**SAS**	**IRN**	**Functional consequence**
***1**	None		83.1	89.8	81.6	99	92.6	95	Normal
***2**	rs11572103	(I269F)*	0.3	0.8	15.2	0	1.9	2.3	Decreased
***3**	rs10509681, rs11572080	(K399R)* (R139K)*	11.2	6.7	2.1	0	4	0	Decreased
***4**	rs1058930	(I264M)*	5.4	2.7	1.1	0	1.5	2.6	Decreased
***5**	rs72558196	Frameshift	0	0	0	0	0	0	Inactive
***6**	rs142886225	(G171S)*	0	0	0	0.5	0	0.1	Normal
***7**	rs72558195	(R186X)**	0	0	0	0	0	0	Inactive
***11**	rs78637571	(E274X)**	0	0	0	0.3	0	0	Inactive
***12**	rs3832694	(461delV)***	0	0	0	0	0	0	Not clear
***14**	rs188934928	(A238P)*	0	0	0	0.2	0	0	Decreased

*: Missense
**: Stop-gain
***: Inframe deletion

Table S4. Important variant and allele frequencies of the human CYP2C9 gene

Important variant and allele frequencies of the human *CYP2C9* gene									
			Allele frequencies in indicated populations, %						
**Allele**	**Defining variants**	**Variant type**	**EUR**	**AMR**	**AFR**	**EAS**	**SAS**	**IRN**	**Functional consequence**
***1**	None		80.2	88.8	79.1	96.3	82.4	78.1	Normal
***2**	rs1799853	(R144C)*	12.6	6.8	2.1	0	4.6	10.5	Decreased
***3**	rs1057910	(I359L)*	6.8	3.8	1.2	3.3	11	10.2	Decreased
***4**	rs56165452	(I359T)*	0	0	0	0	0	0	Decreased
***5**	rs28371686	(D360E)*	0	0	1.1	0	0	0	Decreased
***6**	rs9332131	Frameshift	0	0	1	0	0	0	Inactive
***7**	rs67807361	(L19I)*	0	0	0	0	0	0	Not clear
***8**	rs7900194	(R150H)*	0	0.2	5.9	0	0	0.1	Decreased
***9**	rs2256871	(H251R)*	0	0.2	7.5	0	0	0.3	Normal
***11**	rs28371685	(R335W)*	0.2	0.1	1.9	0	0.2	0.1	Decreased
***12**	rs9332239	(P489S)*	0.2	0.1	0	0	0	0.1	Decreased
***13**	rs72558187	(L90P)*	0	0	0	0.1	0	0	Decreased
***14**	rs72558189	(R125H)*	0	0	0	0	1.8	0.1	Not clear
***15**	rs72558190	(S162X)**	0	0	0	0	0	0	Inactive
***16**	rs72558192	(T299A)*	0	0	0	0.3	0	0	Not clear
***29**	rs182132442	(P279T)*	0	0	0	0.1	0	0.4	Not clear
***30**	rs781583846	(A477T)*	0	0	0	0	0	0	Not clear
***31**	rs57505750	(I327T)*	0	0	0	0	0	0	Decreased
***33**	rs200183364	(R132Q)*	0	0	0	0	0	0.1	Not clear
***36**	rs114071557	Start lost	0	0	0.2	0	0	0	Not clear
***42**	rs12414460	(R124Q)*	0	0	0	0	0	0	Not clear
***44**	rs200965026	(T130M)*	0	0	0	0	0	0	Not clear
***45**	rs199523631	(R132W)*	0	0	0	0	0	0	Not clear

*: Missense

**: Stop-gain

Table S5. Important variant and allele frequencies of the human CYP2C19 gene

**Important variant and allele frequencies of the human *CYP2C19* gene**									
			**Allele frequencies in indicated populations, %**						
**Allele**	**Defining variants**	**Variant type**	**EUR**	**AMR**	**AFR**	**EAS**	**SAS**	**IRN**	**Functional consequence**
***1**	None		62.1	79.1	48.5	62.4	67.3	85.8	Normal
***2**	rs4244285	**	14.6	10.1	17.7	30.7	32.4	13.1	Inactive
***3**	rs4986893	(W212X)#	0	0	0	6.2	0.3	0.3	Inactive
***4**	rs28399504	Start lost	0	0.2	0	0	0	0	Inactive
***5**	rs56337013	(R433W)*	0	0	0	0	0	0	Inactive
***6**	rs72552267	(R132Q)*	0	0	0	0	0	0	Inactive
***7**	rs72558186	**	0	0	0	0	0	0	Inactive
***8**	rs41291556	(W120R)*	0.2	0	0	0	0	0.2	Inactive
***9**	rs17884712	(R144H)*	0	0	1.2	0	0	0	Decreased
***10**	rs6413438	(P227L)*	0	0	0.3	0	0	0	Decreased
***12**	rs55640102	(X491C) ***	0	0	0.1	0	0	0	Not clear
***13**	rs17879685	(R410C)*	0	0	1.7	0	0	0	Normal
***15**	rs17882687	(I19L)*	0	0	1.8	0	0	0.1	Normal
***16**	rs192154563	(R442C)*	0	0	0	0	0	0	Decreased
***17**	rs12248560	Regulatory	23.1	10	20.9	0.7	NA	NA	Increased
***22**	rs140278421	(R186P)*	0	0	0.1	0	0	0	Inactive
***23**	rs118203756	(G91R)*	0	0	0	0	0	0	Not clear
***24**	rs118203757	(R335Q)*	0	0	0	0	0	0.5	Inactive
***25**	rs118203759	(F448L)*	0	0	0	0	0	0	Decreased
***27**	rs7902257	Regulatory	0	0.6	7.7	0	0	0	Decreased

*: Missense
**: Splicing defect
***: Stop-lost
#: Stop-gain
NA: Not Available (Data not found)

Table S6. Important variant and allele frequencies of the human CYP2D6 gene

Important variant and allele frequencies of the human *CYP2D6* gene									
			Allele frequencies in indicated populations, %						
**Allele**	**Defining variants**	**Variant type**	**EUR**	**AMR**	**AFR**	**EAS**	**SAS**	**IRN**	**Functional consequence**
***1**	None		21.9	44.5	14.4	14.1	27.6	10.6	Normal
***1xN**	Amplification of *1		1	0.5	3.3	1	0.5	NA	Increased
***2**	rs16947, rs1135840	(R296C)* (S486T)*	34.3	32.7	26.7	14	36.2	47	Normal
***2xN**	Amplification of *2		1.3	0.5	6	1	1	NA	Increased
***3**	rs35742686	Frameshift	4.7	0.4	0.2	0	0.1	0.3	Inactive
***4**	rs3892097	#	19.6	11.1	8	0.3	10.3	11.2	Inactive
***5**	CYP2D6 deleted		3	3	4	6.5	2	NA	Inactive
***6**	rs5030655	Frameshift	1	0.3	0.2	0	0.1	0.5	Inactive
***7**	rs5030867	(H324P)*	0	0	0	0	0.8	0.3	Inactive
***8**	rs5030865	(G169X)**	0	0	0	0	0	0	Inactive
***9**	rs5030656	(K281del)***	2.5	1.2	0.3	0	0.2	0	Decreased
***10**	rs1065852, rs1135840	(P34S)* (S486T)*	0.2	0	3.2	58.7	6.5	15.1	Decreased
***11**	rs201377835	#	0	0	0	0	0	0	Inactive
***12**	rs5030862	(G42R)*	0	0	0	0	0	0.2	Inactive
***14**	rs5030865	(G169R)*	0	0	0	1.2	0	0	Decreased
***17**	rs16947, rs28371706	(R296C)* (T107I)*	0	0.7	19.7	0	0.1	0	Decreased
***29**	rs16947, rs1135840, rs61736512, rs59421388	(R296C)* (S486T)* (V136I)* (V338M)*	0	0.4	9.2	0	0	0	Decreased
***33**	rs28371717	(A237S)*	1.2	0.1	0.2	0	0.6	0.8	Normal
***41**	rs28371725	#	9.3	4	2.6	3.2	13.5	14	Decreased
***42**	rs72549346	Frameshift	0	0	0.2	0	0	0	Inactive
***43**	rs28371696	(R26H)*	0	0.1	1.8	0	0.5	0	Not clear
***53**	rs1135822, rs1135823	(F120I)* (A122S)*	0	0.5	0	0	0	0	Normal
***62**	rs730882171	(R441C)*	0	0	0	0	0	0	Inactive

*: Missense
**: Stop-gain
***: Inframe deletion
#: Splicing defect
NA: Not Available (Data not found)

Table S7. Important variant and allele frequencies of the human CYP2E1 gene

Important variant and allele frequencies of the human *CYP2E1* gene										
			Allele frequencies in indicated populations, %							
**Allele**	**Defining variants**	**Variant type**	**EUR**	**AMR**	**AFR**	**EAS**	**SAS**	**IRN**	**Functional consequence**
***1**	None		93.5	86.1	79.2	79.4	97.2	94.1	Normal
***2**	rs72559710	(R76H)*	0	0	0	0.4	0	0	Decreased
***3**	rs55897648	(V389I)*	0.2	0	0	0	0	0.3	Normal
***4**	rs6413419	(V179I)*	2.2	2.4	20.6	0	1.9	5.6	Normal
***5**	rs3813867, rs2031920	5' UTR	4.1	11.5	0.2	20.2	0.9	0	Not clear

*: Missense

Table S8. Important variant and allele frequencies of the human CYP3A4 gene

Important variant and allele frequencies of the human *CYP3A4* gene									
			Allele frequencies in indicated populations, %						
**Allele**	**Defining variants**	**Variant type**	**EUR**	**AMR**	**AFR**	**EAS**	**SAS**	**IRN**	**Functional consequence**
***1**	None		94.4	97.2	96.9	97.3	99.8	99.7	Normal
***2**	rs55785340	(S222P)*	0	0	0	0	0	0	Not clear
***3**	rs4986910	(M445T)*	0.7	0.1	0	0	0	0.1	Not clear
***4**	rs55951658	(I118V)*	0	0	0	0.5	0	0	Not clear
***5**	rs55901263	(P218R)*	0	0	0	0.1	0	0	Not clear
***6**	rs4646438	Frameshift	0	0	0	0.2	0	0	Not clear
***7**	rs56324128	(G56D)*	0.1	0	0	0	0	0	Not clear
***8**	rs72552799	(R130Q)*	0.1	0	0	0	0	0	Decreased
***9**	rs72552798	(V170I)*	0	0	0	0	0	0	Not clear
***10**	rs4986908	(D174H)*	0.3	0	0.1	0	0.1	0	Not clear
***11**	rs67784355	(T363M)*	0	0	0	0	0	0.2	Decreased
***12**	rs12721629	(L373F)*	0	0	0.3	0	0	0	Decreased
***13**	rs4986909	(P416L)*	0	0	0	0	0	0	Decreased
***15**	rs4986907	(R162Q)*	0	0.2	2.6	0	0	0	Not clear
***16**	rs12721627	(T185S)*	0	0	0	0	0	0	Decreased
***18**	rs28371759	(L293P)*	0	0	0.1	1.9	0	0	Decreased
***19**	rs4986913	(P467S)*	0	0	0	0	0.1	0	Not clear
***20**	rs67666821	Frameshift	0	0.1	0	0	0	0	Inactive
***22**	rs35599367	Intronic	4.4	2.4	0	0	0	0	Decreased
***26**	rs138105638	(R268X)**	0	0	0	0	0	0	Inactive

*: Missense
**: Stop-gain

Table S9. Important variant and allele frequencies of the human CYP3A5 gene

Important variant and allele frequencies of the human *CYP3A5* gene									
			Allele frequencies in indicated populations, %						
**Allele**	**Defining variants**	**Variant type**	**EUR**	**AMR**	**AFR**	**EAS**	**SAS**	**IRN**	**Functional consequence**
***1**	None		6.4	20.6	45.9	27.1	31.2	3.8	Normal
***2**	rs28365083	(T398N)*	0.6	0.1	0.1	0	0	0	Not clear
***3**	rs776746	#	93	78.2	31	71.8	68.8	96.1	Inactive
***4**	rs56411402	(Q200R)*	0	0	0	0.5	0	0	Not clear
***5**	rs55965422	#	0	0	0	0.6	0	0	Not clear
***6**	rs10264272	#	0	0.7	12.8	0	0	0.1	Inactive
***7**	rs41303343	Frameshift	0	0.4	10.2	0	0	0	Not clear
***8**	rs55817950	(R28C)*	0	0	0	0	0	0	Decreased

*: Missense

#: Splicing defect
